# A Comparative Study of Nesting Sites and Burrowing Habits of Two Iranian Burrowing Scorpions

**Published:** 2017-03-14

**Authors:** Babak Vazirianzadeh, Amir Jalali, Mostafa Chrom, Alireza Mohammady, Hassan Vatandoost, Forozan Panahi

**Affiliations:** 1 Social Determinants of Health Research Center, Ahvaz Jundishapur University of Medical Sciences, Ahvaz, Iran; 2 Depatrment of Medical Entomology, Ahvaz Jundishapur University of Medical Sciences, Ahvaz, Iran; 3 Depatrment of Pharmacology and Toxicology, School of Pharmacy and Toxicology Research Centre, Ahvaz Jundishapur University of Medical Sciences, Ahvaz, Iran; 4 Depatrment of Soil Science, School of Agriculture, Ahvaz University of Shahid Chamran, Ahvaz, Iran; 5 Depatrment of Medical Entomology, Ahvaz Jundishapur University of Medical Sciences, Ahvaz, Iran; 6 Depatrment of Medical Entomology and Vector Control, School of Public Health and National Institute of Health Research, Tehran University of Medical Sciences, Tehran, Iran

**Keywords:** Nesting sites, *Odonthubutus bidentatus*, *Scorpio maurus*, Scorpions, Habits

## Abstract

**Background:**

The different features of scorpions can be successfully described by their nesting and burrowing behaviors. There is little information about burrowing activity of Iranian scorpions.

**Methods:**

The current study was performed to compare the burrowing behavior between two burrowing Iranian scorpions, *Scorpio maurus* and *Odonthubutus bidentatus* by describing 30 nests of each species regarding collecting the scorpions.

**Results:**

*Scorpio maurus* and *O. bidentatus* have a tendency to make nest with elliptical, round-like entrance and oval shape with arch at the top, respectively. There was not any significant difference between nest entrance properties of two scorpions. One-way ANOVA test showed that the height and diameter of two species nests were not significantly different. A Pearson correlation also showed a relative strong direct relationship between height and diameter of *S. maurus* nests than *O. bidentatus*. This correlation was not significant in the case of *O. bidentatus*. The results provided additional habitat information of scorpions.

**Conclusion:**

The nests morphology characteristics of two Iranian scorpions including shape, depth, length and diameter depend are different from each other based on the following factors: species, soil texture, soil moisture and region conditions.

## Introduction

Scorpions are terrestrial arthropods with generally non-social habits. The majority of scorpions are nocturnal, particularly species inhabiting arid desert regions. The various degrees of adaptation to different ecological conditions have been reported for scorpions (Prendni and Wheeler 2005, Fet and Soleglad 2005, Cala-Riquelme and Colombo 2010). Therefore, this group of animals is subject to different investigations concerning their ecological, physiological and biochemical adaptations in adjusting to harsh conditions found in their habitats.

In spite of many different ecological and biological aspects regarding to scorpions, there are a little information in this regard. One of these ecological aspects, not known properly, is burrowing habit. Burrowing habit provide important facilities for scorpions such as protection against predation, increased availability of food and ideal microclimate for their lives (Shehab 2011, Hembree et al. 2012).

Scorpions live under rocks, logs and plants during the day or in cracks and burrow their shelters and nests (Cala-Riquelme and Colombo 2010). “Normally, a majority of scorpion species are able to make some changes in their places and less able to make their nests through burrowing” (Cala-Riquelme and Colombo 2010, 2000, Shehab 2011).

There are six burrowing scorpion species in Iran including *S. maurus* (Scorpionida: Scopionidae), *O. bidentatus*, *O. doriae*, *O. odonthodorous*, *Apisthobothus petrigosercus* and *A. susanei* (Scorpionida: Buthidae) (Farzanpey 1987, Navidpour 2008).

*Scorpio maurus* is seen generally in plains close to wheat fields or in deep holes of deserts. This specie has been recorded in different Iranian provinces such as Khuzestan, Kurdistan, Fars, Azarbaijan, Qazvin, Isfahan and Bushehr (Farzanpey 1988, Dehghani 1991).

*Odonthubutus bidentatus*, is another digger scorpion found mostly in the desert and low altitude regions. This species was formerly called *O. odonturus* (Farzanpey 1987). The geographical distribution showed that *O. bidentatus* is restricted to the southwest regions of Iran, mainly Khuzestan. This species is not well investigated but in recent years, more investigations were focused on the other members of the genus, *O. doriae* (Dehghani et al. 1991, Jalali et al. 2007, Navidpour 2008, Masihipour et al. 2009, Jalali et al. 2010). This scorpion, distributed in Khuzestan, has potentially been considered as one of the medically important scorpion in the region. However, its sting potency is roughly equivalent to that of *O. doriae*, with a decreased risk for significant neuromuscular compromise. The Haftgel area is recognized in terms of having high frequency of *S. maurus* and *O. bidentatus* in south west of Iran, Khuzestan (Farzanpey 1987, Vazirianzadeh and Samie 2006). Therefore, this area was selected as an appropriate area for this study.

The purpose of this study was to investigate and provide more information regarding soil texture and soil relative humidity (sutured percentage), identification and drawing the patterns of the nest making habits and nesting activities of two Iranian burrowing scorpions. This issue is an important help to provide the suitable life conditions of both species in laboratory and scorpion breeding studies in future researches.

## Materials and Methods

### Sampling and trapping

The scorpion samples were collected initially from Haftgel (31° 26′ 35.85″ N, 49° 31′ 38.93″ E). The scorpions were selected through a particular nest sampling procedure. Firstly, the elliptical or round holes were recognized in none resident fields of Haftgel as nests of scorpions. Then the nests were filled with a calcium carbonate suspension in order to move scorpion into the opening and solid formation the way of nest. Next to scorpion observation, the back entrance of the nest was blocked and scorpion trapped in particular glasses (open door and porous). The scorpion samples were transferred to medical entomology laboratory, Department of Medical Entomology, School of Public Health, Ahvaz Jundishapur University of Medical Sciences (AJUMS), Ahvaz, Iran and identified according to common Iranian scorpion keys (Farzanpey 1991, Navidpour et al. 2008). Thirty nests of each species were observed during this study, which dimensions of them were measured.

### Determination of Physical structure and texture of soil

In order to determine the physical structure and type of soil texture, the sampling was carried out randomly in the ranges of 10–20, 20–40 and 40–60cm deep of the nests from two places. This sampling was performed using excavator cylinders (Oger®). The properties of soil samples including type of soil, percentage of ingredients and moisture content of different depths were identified using hyderometery mechanical analysis procedure (Day 1982). This identification was performed in the Soil Science Department, School of Agriculture, Shahid Chamran University, Ahvaz, Iran.

### Nests pattern identification

In order to measure and draw the picture of the nest tunnels and determination and preventing of soil texture disassembling throughout the tunnel route, a molage method with masonry plaster modeling was used. After initial recognition, half to one-liter slurry solution with usual viscosity was used for each nest. Subsequently, the entrance of the nest at ground was turned into cone-shape with around soil. The gypsum solution was poured slowly in a stage into the entrance of nest. The aforesaid formats were firmed and molage was made after 30 to 60min. Molage dimensions were measured and obtained patterns were drawn. Furthermore, the diameter and heights of nests were measured by a caliber. The density of distribution nests was determined using two ways: 1) using the number of nests in a 1m^2^ wooden quadrant for each species Ten quadrants were applied in the each habitats belonged to each species in two different sites where the definite scorpions were collected. 2) Numbers of counted nests in five min (same as above aforementioned procedure).

### Statistical analysis

Statistical analysis between nests was carried out by using one way ANOVA and Pearson correlation. Minitab13 statistical software was used to determine the nests characteristics and relations between two species.

## Results

Both species were collected from same parts of the studied field. Mechanical analysis (Hydrometery tests) of examined soils indicated that the type of textures in the 10–20, 20–40cm and 40–60cm depths of scorpion nests were LOAM, SILT-LOAM and LOAM, respectively. Hydrometery tests evaluated the contents of humidity of soil (sutured percentage) as 32.11%, 37.93% and 36.34% in the 10–20, 20–40 and 40–60cm depths of the nests, respectively. The structure of the studied area in the 60 cm depth was calcareous and rocky sections. The results are performed in the Table 1.

**Table 1.** Soil texture and Physical properties of Haftgel area, 2010

**Depth of soil (cm)**	**Soil class**	**% of loam**	**% of clay**	**% of sand**	**% of sutured humidity**
**10–20**	LOAM	48	13	39	32.11
**20–40**	SILT.LOAM	50	23	27	37.93
**40–60**	LOAM	47	18	35	36.34

Diameter and height variables of *S. maurus* and *O. bidentatus* nests openings are presented in Table 2 and 3, respectively. The means of S*. maurus* and *O. bidentatus* height and diameter nests were determined as 1.08± 0.31 and 1.02±0.25cm and 1.78±0.42 and 1.8±0.4cm, respectively. In addition, one-way ANOVA test showed that the height and diameter of two species nests were not significantly different (P> 0.05). Pearson correlation also showed a relative strong direct relationship between height and diameter of *S. maurus* nests (r= 0.67). This correlation was not a strong significant in the case of *O. bidentatus* (r= 0.43).

**Table 2.** Inlet height and diameter of *Scorpio maurus* nests

**Height of nest**	**Diameter of nest**	**Height of nest**	**Diameter of nest**	**Height of nest**	**Diameter of nest**
1.38	1.94	1.59	2.08	0.97	1.97
0.67	1.14	0.98	1.71	1.12	2.31
0.83	1.72	1.12	1.68	1.19	1.79
0.79	1.19	1.29	1.86	0.94	1.33
1.38	2.19	0.95	1.34	0.71	1.18
0.87	1.76	1.05	1.89	1.53	2.29
1.39	2.1	0.81	1.98	0.44	0.92
1.22	2.19	0.83	1.21	1.96	2.15
1.19	1.34	1.12	2.33	1.21	2.33
1.22	2.33	1.09	1.48	0.85	1.91

**Table 3.** Inlet height and diameter of *Odonthubutus bidentatus* nests

**Diameter of nest**	**Height of nest**	**Diameter of nest**	**Height of nest**	**Diameter of nest**	**Height of nest**
1.24	2.18	1.07	2.22	1.32	1.93
1.10	2.28	0.50	1.11	1.12	1.82
1.14	1.50	0.77	1.53	1.08	2.08
1.14	2.03	1.24	1.82	1.38	2.13
1.40	1.73	0.87	1.16	1.14	2.10
0.94	1.72	1.14	1.16	1.06	2.27
0.90	1.52	0.76	1.76	1.35	2.16
0.52	2.62	0.77	1.34	0.61	1.60
1.16	1.85	1.46	2.50	0.7	1.26
1.04	2.04	1.05	1.53	0.82	1.34

**Fig. 1.**
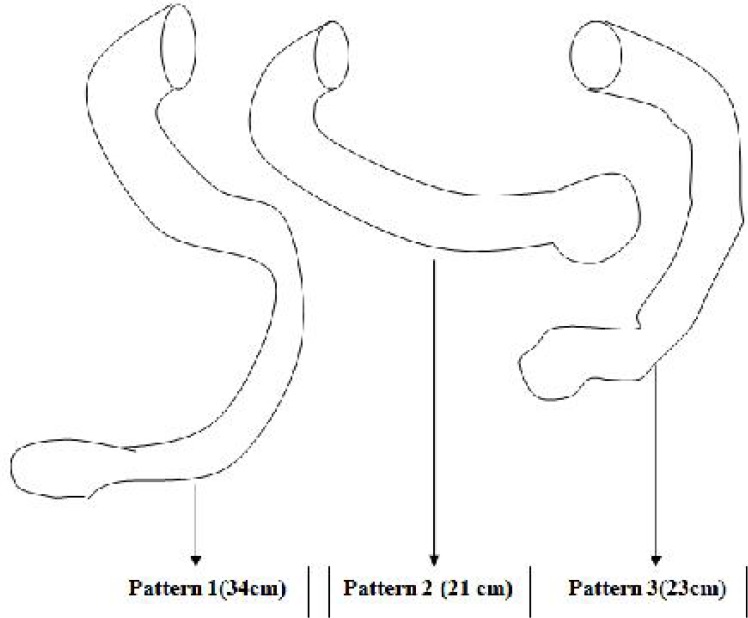
Patterns of *Scorpio maurus* nests in Haftgel, south west of Iran (original picture)

*Scorpio maurus* has a tendency to make nest with elliptical and round-like entrance. Their nests are began with more or less a sharp slope and then screw in the opposite direction to end finally to a sloping horizontal. *Scorpio maurus* lives at the end of the nest. The length of *S. maurus* nests were measured 21–34cm (three nests were dissected), approximately (Fig. 1).

**Fig. 2.**
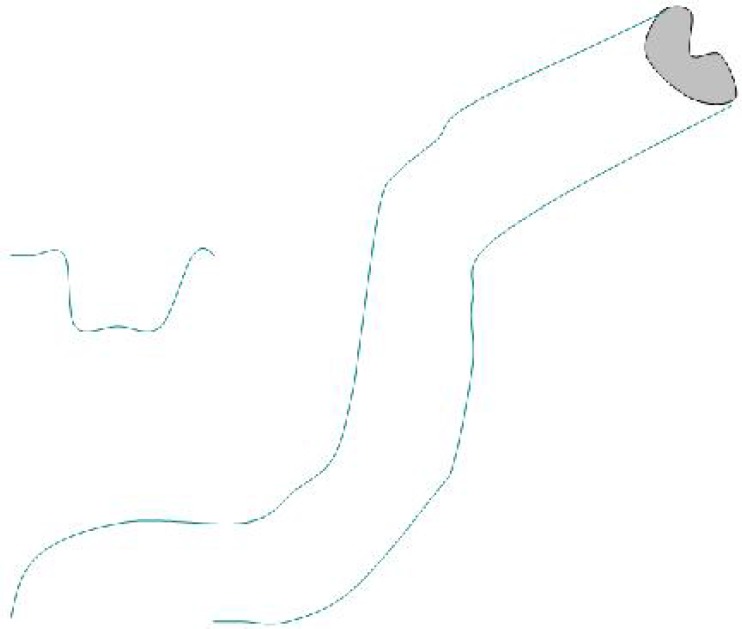
Patterns of *Odonthubutus bidentatus* nests (35–55cm) in Haftgel, south west of Iran (original picture)

*Odonthubuthus bidentatus* nests had oval shape with an arch at the top. The nest was built first with a slow slope and then followed with deeper degree and finally completely became horizontal until to end. The length of *O. bidentatus* nests was measured 35–55cm, approximately (Fig. 2) (5 nests were dissected).

The density of nests in the studied area was measured 0.4 nests of *S. maurus* and 0.6 nests of *O. bidentatus* in 1m^2^ and counted 19 nests in 5 min related to both species. The results also opined that the majority of nests are in a spiral manner special regarding *S. maurus* nests.

The entrapping was done in the early morning when the scorpions more movement and activity to come back to their nests after night promenade. The majority of *S. maurus* scorpions were inside of their nests (75% of trapped scorpions, or 22 *S. maurus* were trapped). The observations showed that the 60% of *O. bidentatus* nests were empty at the entrapping times (12 *O. bidentatus* were trapped). Totally 30 nests of each species were observed. The molage procedure was performed for 3 nests of *S. maurus* and 5 nests of *O. bidentatus*.

Pictures of all 3 *S. maurus* nests are performed as Fig. 1 and only 1 picture of *O. bidentatus* is performed as Fig. 2 because the 5 nests were in very similar shape and way.

## Discussion

The Iranian scorpions are classified into 3 categories of non-burrowing (*Hemiscorpius lepturus*), semi-burrowing (*Mesobuthus eupeus* and *Andrectonus crassicauda*) and burrowing scorpions (*S. maurus*, *O. Bidentatos*, *O. odonthodorous*, *A. petrigosecus* and *O. doriae*) according to the pattern of nest building habits (Vazirianzadeh and Tirgari 1989). This paper describes the architectural and surficial morphologies of burrows produced by *S. maurus* as well as *O. bidentatos*. This study was carried out in order to identify the burrowing habitat, nesting features and then linked to environmental conditions including soil composition, density and moisture content.

The type of soil texture in the area of study was determined as a light class of soil including clay loam and sandy loam. This is in line with this fact that the high density of nests was related to the lightness of soil texture (Rutin 1996). The moisture (sutured humidity) was raised from 32.11% in 10–20cm to 37.93% in the depth of 20–40cm due to raising the clay content from 13% to 23%. This finding is in consent to previous study in terms of *C. jonesii* burrowing habit regarding increasing clay content in the soil structure (Harington et al. 1978). This may explain that deep of nests in the study area usually was distributed in 20–35cm depth range regarding both species. Burrow depth approaching 75cm have been reported for *S. maurus* in the Sahara Desert (Cloudsley-Thompson 1965). This implies that nests are not very deep in the calcareous and rocky sections and the drilling attempts had been stopped at the current depths of 20–35cm (Rutin 1996). The depth of *O. doriae* nest has been reported 20.5±5.5cm (Dehghani et al. 1991). It means that burrowing habits is different even between two species of one genus. However, this may be due to the soil structure of the region of study.

The density of nests area was measured 0.4 nests in 1m^2^ related to *S. maurus* or counted 19 nests in 5 min (regardless of species) and this rate was 0.6 regarding to *O. bidentatus*. Consequently, the soil texture of this area may provide more facilities for drilling activity of *O. bidentatus* than *S. maurus* but the number of trapped the latter species was greater. It is presumed that there were more factors determined the density values of both species including size of scorpion bodies and shape of their pedipalps or other drilling organs.

This is in accordance with Hadley (1974) and Hembree et al. (2012), who explained that burrowing habit in scorpions was closely related to their taxonomic position and morphological modifications. The nests of *S. maurus* had been built using more engineer methods with more spirals in the shapes than nests of *O. bidentatus*, in the current study.

Any artificial breeding activity for each of these species in natural enclosed piece of land, that may be called scorpion Zoo Park, need to be created separately with particular structure of soil texture. However, in the current case this is an advantage which both species can live in an area with the same soil texture.

Khuzestan Province is a desert and plain region with a semi-arid climate and almost no raining occur from mid or late of May to end of September (Vazirianzadeh 1989). The soil texture nests of both species were sandy loam in 10–20cm depth and clay loam in 20–40cm depth. The soil of area had gypsum normally formed following raining. Therefore, this condition made to reduce the chance of survival a burrowing than a non-burrowing scorpion in this province. Due to the changing weather conditions with dry, warm days and cold nights, nests were often made spiral shape. This condition had caused nests built with multi steps. Each step was used by the scorpions to do their rests at a special time due to the different conditions during 24 h (Hadley 1974).

In the term of entrapping in the early morning, acquired results showed that *O. bidentatus* scorpions were engaged in hunting activities, in contrast to the *S. maurus* scorpions, which were present at their nests at the same time. Therefore, human contact with *O. bidentatus* was most likely than *S. maurus* in the early morning. Furthermore, the majority of *O. bidentatus* nests was seen in intact areas far from residential region and was less observed by the residents than *S. maurus* scorpions. Among 990 envenomed individuals by scorpion stings in Khuzestan, 0.4% and 0.1% of cases were related to *S. maurus* and *O. bidentatus*, respectively (Vazirianzadeh and Samie 2006)

Distribution and profiles of nests are presented in Table 1 and 2. The entries show a round hollow nest belongs to *S. maurus*. *Odonthubutus bidentatus* nests were equipped with an elliptical hollow and a small arch in the upper edge. A single elliptical surface opening leads to a single narrow and flat tunnel that ends in a vertically expanded chamber. This instruction plays important role in creating awareness and precautions about scorpion nests in human communities. Education also helps human to understand where to access dangerous scorpion as well as how to escape them.

Although there was not any significant different between nest entrance properties, stronger correlation between nest entrance properties of *S. maurus* than *O. bidentatus* was seen. This correlation may be assumed as a different manner of burrowing habitats between two species in the current study. However, different nest entrance properties between two species, individually, may be implicated as a presentation of different age sizes. This is a positive phenomenon in growing two species of scorpions in scorpion zoo parks. This adaptation makes possibility to build artificial nests in provided area, because interrupt different generation shows that parent and offspring scorpions can tolerate each other.

Therefore, it is taken that the burrow of *S. maurus* was more complex than the typical burrow of O*. bidentatus* and the nests morphology (including shape, depth, length and diameter) depends on the following factors: species, soil texture, soil moisture and region weather conditions (Hembree et al. 2012). Finally, there were some differences between two mentioned scorpions in burrowing activities.

The difference between the 2^nd^ and the 3^rd^ patterns of *S. maurus* is on the depth of the burrowing activities. The presented fact related to the humidity rate of soil, which caused to create different pattern of burrowing activity, by the scorpion is the different between 2^nd^ and 3^rd^ patterns of *S. maurus*. The depth of 2^nd^ pattern was shorter than the depth of the 3^rd^ pattern. In the 2^nd^ pattern the scorpions had reached in the lesser depth to enough soil humidity, then the depth of 2^nd^ pattern was shorter than the two other patterns.

## Conclusion

The nests morphology characteristics of two Iranian scorpions including shape, depth, length and diameter depend are different from each other based on the following factors: species, soil texture, soil moisture and region conditions.
